# Retrospective Evaluation of Hairy Cell Leukemia Patients Treated with Three Different First-Line Treatment Modalities in the Last Two Decades: A Single-Center Experience

**DOI:** 10.4274/tjh.2016.0443

**Published:** 2017-12-01

**Authors:** Şeniz Öngören, Ahmet Emre Eşkazan, Selin Berk, Tuğrul Elverdi, Ayşe Salihoğlu, Muhlis Cem Ar, Zafer Başlar, Yıldız Aydın, Nükhet Tüzüner, Teoman Soysal

**Affiliations:** 1 İstanbul University Cerrahpaşa Faculty of Medicine, Department of Hematology, İstanbul, Turkey; 2 İstanbul University Cerrahpaşa Faculty of Medicine, Department of Pathology, İstanbul, Turkey

**Keywords:** Cladribine, Hairy cell leukemia, Interferon, Splenectomy

## Abstract

**Objective:**

In this study, we retrospectively analyzed the clinical outcome, treatment responses, infectious complications, and survival rates of 71 hairy cell leukemia (HCL) cases.

**Materials and Methods:**

Sixty-seven patients received a first-line treatment and 2-chlorodeoxyadenosine (cladribine-2-CdA) was administered in 31 cases, 19 patients received interferon-alpha (INF-α), splenectomy was performed in 16 cases, and rituximab was used in one.

**Results:**

Although the highest overall response rate (ORR) was observed in patients receiving 2-CdA upfront, ORRs were comparable in the 2-CdA, INF-α, and splenectomy subgroups. Relapse rates were significantly lower in patients who received first-line 2-CdA. The progression-free survival (PFS) rate with 2-CdA was significantly higher than in patients with INF-α and splenectomy, but we found similar overall survival rates with all three upfront treatment modalities. Infections including tuberculosis were a major problem.

**Conclusion:**

Although purine analogues have improved the ORRs and PFS, there is still much progress to make with regard to overall survival and relapsed/refractory disease in patients with HCL.

## Abstract (tr)

**Amaç:**

Bu çalışmada, geriye dönük olarak 71 tüylü hücreli lösemi (SHL) olgusunu klinik sonuçlar, tedavi yanıtları, enfeksiyon komplikasyonları ve sağkalım oranları açısından analiz ettik.

**Gereç ve Yöntemler:**

Altmış yedi hasta birinci basamak tedavi almıştı ve 2-klorodeoksiadenozin (kladribin-2-CdA) 31 olguya uygulanmış, 19 hasta interferon-alfa (INF-α) almış, splenektomi 16 olguda uygulanmış ve rituksimab ise bir hastada kullanılmıştı.

**Bulgular:**

En yüksek toplam yanıt oranı (TYO) birinci basamak 2-CdA alan hastalarda görülmüş olsa da, TYO’lar 2-CdA, INF-α ve splenektomi alt gruplarında benzerdi. Nüks oranları birinci basamak 2-CdA alan hastalarda anlamlı olarak daha azdı. Progresyonsuz sağkalım (PS) oranı 2-CdA alanlarda INF-α ve splenektomi hastalarına göre anlamlı olarak daha yüksek olmakla birlikte, her üç birinci basamak tedavi yaklaşımı ile toplam sağkalım (TS) oranları benzer olarak bulundu. Tüberkülozun da dahil olduğu enfeksiyonlar önemli bir problemdi.

**Sonuç:**

Her ne kadar pürin analogları TYO ve PS’yi iyileştirmiş olsa da, SHL hastalarında TS ve nüks/dirençli hastalık açısından daha yapılması gereken çok şey vardır.

## INTRODUCTION

Hairy cell leukemia (HCL) is a rare mature B-cell neoplasm characterized by the accumulation of atypical lymphocytes with prominent cytoplasmic projections in the bone marrow and spleen, resulting in pancytopenia and splenomegaly [1,2]. Most patients eventually require therapy owing to worsening cytopenias, frequent and life-threatening infections, and/or symptomatic splenomegaly. Several treatment modalities, including splenectomy and immunotherapy with interferon-alpha (INF-α), were used with various clinical and hematologic responses until the introduction of the purine nucleoside analogues 2-chlorodeoxyadenosine (cladribine, 2-CdA) and 2′-deoxycoformycin (pentostatin) [3,4,5]. Purine analogues have resulted in higher complete response (CR) rates and durable remissions, and they have become the treatment of choice in most cases [6]. Monoclonal antibodies (i.e. rituximab) and immunotoxins are currently recommended for relapsed/refractory (R/R) cases [7,8,9]. Also among the target-oriented therapeutic options, the BRAF inhibitor vemurafenib can be used in patients with R/R HCL [10,11].

In this study, we retrospectively analyzed the clinical outcome, treatment responses, infectious complications, and survival rates of HCL patients treated in our institution with three treatment modalities (splenectomy, INF-α, and 2-CdA) as first-line therapy between 1991 and 2014.

## MATERIALS AND METHODS

### Patients

A total of 71 patients with HCL, who were diagnosed and followed in our clinic over the past 20 years, were included in this study. Diagnosis of HCL was established by morphological, flow-cytometric, and immunohistochemical analysis of peripheral blood, bone marrow, and/or spleen specimens. Information on the patients’ characteristics, presenting signs and symptoms, treatment modalities and outcomes, and infections were retrospectively taken from the patients’ files. While defining the patient cohort, we excluded cases with variant HCL.

### Treatment Modalities

Patients were divided into 3 subgroups according to the first-line treatments (i.e. splenectomy, INF-α, and 2-CdA) that they had received. Splenectomy was performed either laparoscopically or via open surgery. 2-CdA was given either by continuous intravenous infusion at a dose of 0.1 mg/kg/day over 7 consecutive days, or by 2-h intravenous infusion at a dose of 0.1 mg/kg once a week for 7 consecutive weeks, depending on whether the patients received it as an inpatient or outpatient treatment, respectively. While on 2-CdA, the patients were given cotrimoxazole prophylaxis against Pneumocystis jirovecii pneumonia. INF-α was administered subcutaneously at a starting dose of 3 MU 3 times a week and maintained with subsequent toxicity-based dose adjustments. Rituximab was administered at the conventional dose of 375 mg/m^2^ weekly for 4 consecutive weeks, as suggested before [7].

### Definition of Response and Survival

Response to treatment was assessed using the criteria described in the consensus resolution of 1987 [12]. Accordingly, CR was defined as the morphological absence of hairy cells (HCs) in the blood and the bone marrow in addition to complete disappearance of hepatosplenomegaly and cytopenias. Normalization of peripheral blood counts together with an at least 50% reduction in the size of organomegaly and the volume of bone marrow HCs, plus <5% circulating HCs, was designated as partial response (PR). Presence of CR or PR was defined as overall response (OR), and any response other than a CR or PR was considered as no response. During response evaluation, a bone marrow biopsy was performed 3 months after finishing 2-CdA treatment. Furthermore, relapse after CR was defined as the reappearance of HCs in the peripheral blood or bone marrow, development of cytopenias, and/or splenomegaly on physical examination. Relapse after PR indicated a >50% increase of residual disease.

Overall survival (OS) denotes the time from the first treatment until the time of death or last follow-up. The duration of progression-free survival (PFS) was calculated from the onset of any first-line treatment until the date of progression. Time to next treatment (TTNT) was calculated as the time from the end of the previous treatment to the institution of the next therapy.

### Statistical Analysis

Student’s t-test was used for the comparison of the quantitative variables. Qualitative variables between groups were compared using the chi-square test. The Kaplan-Meier method was used for survival analysis [13]. Survival rates were compared by using the log-rank test. All tests were two-sided, and p<0.05 was considered as statistically significant. All analyses were performed with SPSS 13.0 for Windows (SPSS Inc., Chicago, IL, USA).

## RESULTS

Sixty-two patients (87%) were male and the median age was 49 years (range: 31-76 years). There were 53 patients (75%) with splenomegaly, and the numbers of patients with hepatomegaly and lymphadenopathy were 32 (45%) and 27 (38%), respectively. The demographic features of the entire cohort including median leukocyte and platelet counts and hemoglobin levels at diagnosis are displayed in Table 1. The median duration of follow-up was 57 months (range: 1-217 months). At diagnosis, all patients had bone marrow biopsy, and flow cytometric evaluation was performed for 47 patients (66%) from peripheral blood and/or bone marrow. At diagnosis, hematoxylin and eosin staining, reticulin staining, tartrate-resistant acid phosphatase staining, and immunohistochemistry for CD20 and annexin A1 were performed from the bone marrow aspiration and trephine biopsy.

### Treatments and Outcomes

**Figure 1:**
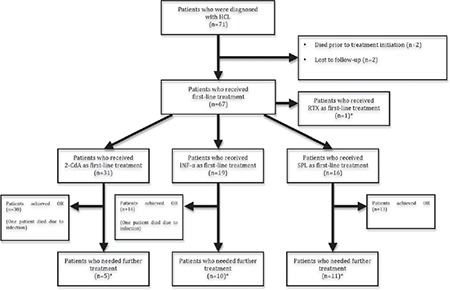
The distribution of first-line treatment modalities and outcomes. 2-CdA: Cladribine, HCL: hairy cell leukemia, INF-α: interferon-alpha, RTX: rituximab, SPL: splenectomy, ORR: overall response rate, NRR: non-response rate. *See text for details.

Among the patient cohort, there were 4 patients who did not receive treatment. Two of them were lost to follow-up, one patient died due to severe infection, and one had an acute myocardial infarction (AMI) and died before any treatment was initiated (Figure 1).

### First-Line Treatment

**Figure 2:**
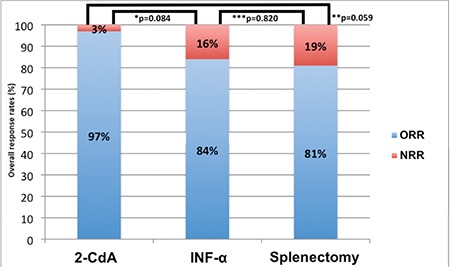
Response rates according to the different first-line treatment modalities. *2-CdA vs. INF-α; **2-CdA vs. splenectomy; ***INF-α vs. splenectomy. ORR: Overall response rate, NRR: non-response rate, 2-CdA: cladribine, INF-α: interferon-alpha.

Sixty-seven (94%) patients received a first-line treatment. 2-CdA was administered for 31 patients (46%), 19 patients (28%) received INF-α, splenectomy was performed in 16 cases (24%), and rituximab was used in one case (2%) (Figure 1). Although patients in the splenectomy arm were younger than those in the other 2 arms, the differences were not statistically significant (Table 2). The 3 treatment subgroups were equally balanced regarding sex distribution, median hemoglobin levels, and leukocyte and platelet counts (Table 2). There were 30, 16, and 13 patients who achieved OR after the first-line treatment with 2-CdA, INF-α, and splenectomy, respectively (Figure 1). The OR rates (ORRs) in the 2-CdA, INF-α, and splenectomy subgroups were 97%, 84%, and 81%, respectively, and although the ORR of 2-CdA treatment was superior to those of the other 2 groups, the differences were not significant (Figure 2). During the first-line treatment, 2 patients (one on 2-CdA and one on INF-α) died due to infection (Figure 1). No postoperative complications were observed in patients with splenectomy.

Five of the 31 patients who received 2-CdA as the first-line therapy required a second-line treatment after a median TTNT of 23 months (range: 3-58 months) (Table 2). There were 10 patients in the first-line INF-α subgroup who progressed and needed a second-line treatment following a median TTNT of 21 months (range: 1-96 months). Eleven of the 16 patients who underwent first-line splenectomy received a second-line therapy due to relapsed disease at a median TTNT of 5 months (range: 2-73 months). Relapse rates were significantly lower in patients who received first-line 2-CdA than those who were treated with INF-α or splenectomy (p=0.007 and p<0.0001, respectively). However, this was not significantly different when the INF-α and splenectomy subgroups were compared (p=0.339) (Table 2). Although the patients with first-line splenectomy had a shorter median TTNT than those with 2-CdA and INF-α, the difference did not reach statistical significance (Table 2). The only patient who received rituximab as frontline treatment remained refractory to that therapy, and she needed further treatment.

### Second-Line Treatment

With a median TTNT of 13 months (range: 1-96 months), 27 patients required a second-line treatment due to R/R disease. Twelve patients received INF-α, 11 patients were treated with 2-CdA, 3 patients underwent splenectomy, and one received fludarabine.

Eight and 2 of the 12 patients who received second-line INF-α had been initially treated with splenectomy and 2-CdA, respectively. In the remaining 2 patients the previous treatment was also INF-α. The ORR was 100% in patients who received second-line INF-α, with only 3 patients requiring a third-line treatment due to relapsed disease.

In patients who had 2-CdA as a second-line treatment, 7 had received INF-α as the first-line therapy, and 2 had splenectomy. The other 2 had been treated with rituximab and 2-CdA previously. All the patients responded to second-line 2-CdA (the ORR was 100%), and only one relapsed after a follow-up of 48 months.

Splenectomy was performed in 3 patients as a second-line treatment. All of them initially responded to splenectomy; however, 2 needed further treatment due to relapsed disease. The patient who received fludarabine as a second-line therapy achieved PR but was then lost to follow-up after 9 months.

### Third-Line Treatment

There were 6 patients who needed third-line treatment. INF-α, 2-CdA, and rituximab were used for 3, 2, and one of them, respectively, following a median TTNT of 36 months (range: 8-63 months). Among patients receiving third-line INF-α, one achieved and maintained CR throughout the entire follow-up. The other 2 remained refractory to INF-α treatment; one died, and the other patient proceeded to fourth-line treatment. One of the 2 patients who received 2-CdA as a third-line treatment achieved and maintained CR and the other patient died due to refractory disease.

The patient who received third-line weekly rituximab could not complete the 4^th^ week of treatment due to an allergic reaction, which had happened following the 3^rd^ dose of the drug, and had to proceed to fourth-line therapy.

### Fourth-Line Treatment

There were only 2 patients who received a fourth-line treatment. One of them was treated with INF-α and the other with rituximab. The patient who received INF-α died due to refractory disease, and the patient who was given rituximab achieved CR.

### Infections

In 47 patients, 76 infectious episodes were noted during the entire follow-up period (Table 3). Bacterial infections were the leading cause, and 64 bacterial infection episodes were observed. Of these 65 episodes, 14 were observed after diagnosis prior to the initiation of any anti-HCL treatment, whereas 50 episodes were noted during treatment (Table 3). The infections were diagnosed by means of cultures, radiological imaging techniques, and tissue biopsies as indicated. There were 18 patients who required hospitalization (mostly due to neutropenic fever), and in 17 cases infections occurred within 30 days after completion of treatment (all patients received 2-CdA).

There were 7 infection episodes caused by Mycobacterium tuberculosis in 6 patients (5 pulmonary cases, 2 disseminated). In 2 patients tuberculosis was diagnosed synchronously with HCL, and 5 episodes occurred during/after anti-HCL treatment. In patients for whom tuberculosis was diagnosed prior to treatment initiation, one had pulmonary tuberculosis and he experienced disseminated disease 3 months after completing 2-CdA. The other patient had disseminated tuberculosis at diagnosis, which was diagnosed via splenectomy. That patient then received INF-α due to relapse without any recurrence of tuberculosis. None of the patients with tuberculosis had drug-resistant disease, and none of them had any known comorbidities including diabetes. There were also 2 patients with invasive pulmonary aspergillosis (IPA).

Six of the 10 episodes of viral infections were related to flu. One of these patients had influenza A virus subtype H1N1 infection (swine flu) and had to be followed in the intensive care unit.

Overall, 3 patients died due to infection, and one of them died because of a febrile neutropenic episode prior to treatment initiation. In the remaining 2 cases, infections occurred during/after treatment; IPA was the reason for death in one patient receiving first-line INF-α and the other patient died due to sepsis following frontline 2-CdA administration. The distribution of the infections and outcomes are shown in Table 3.

### Survival

**Figure 3:**
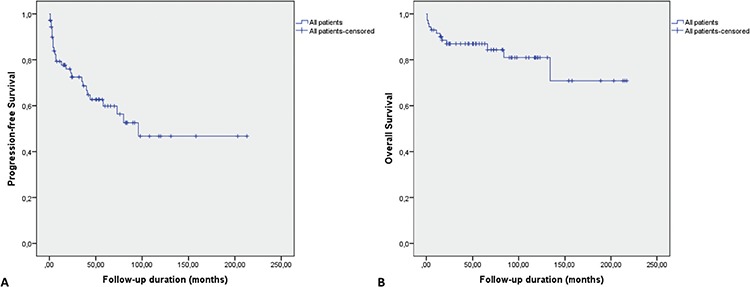
The progression-free survival (A) and overall survival (B) of the entire cohort. 2-CdA: Cladribine, INF-α: interferon-alpha.

**Figure 4:**
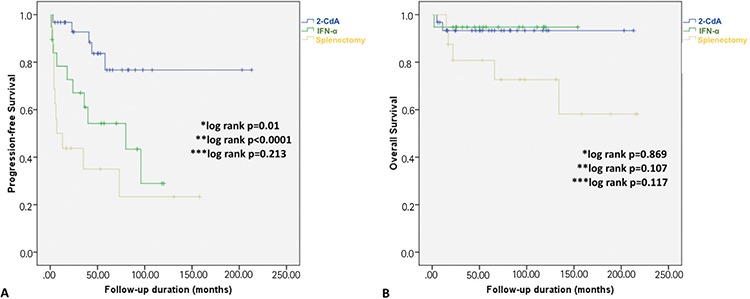
The progression-free survival (A) and overall survival (B) when patients were divided into 3 groups according to the first-line treatment. *2-CdA vs. INF-α; **2-CdA vs. splenectomy; ***INF-α vs. splenectomy. 2-CdA: Cladribine, INF-α: interferon-alpha.

During the follow-up, 10 patients (12%) died (7 due to refractory disease and/or infections, 3 due to AMI and sudden cardiac death) and 2 patients were lost to follow-up. PFS and OS rates for the entire cohort after a median follow-up of 57 months were 62% (Figure 3B) and 83% (Figure 3B), respectively. With regard to the first-line treatment, the PFS rate was significantly higher for patients who received 2-CdA than those for patients who were treated with INF-α and splenectomy (p=0.01 and p<0.0001, respectively). However, PFS did not statistically differ between patients who were treated with INF-α and those who underwent splenectomy (p=0.213) (Figure 4A). There was no statistically significant OS difference between these 3 treatment modalities, although survival rates achieved with first-line 2-CdA and INF-α seemed to be superior to those achieved with splenectomy (Figure 4B).

## DISCUSSION

In this study we retrospectively evaluated the demographic features of patients with HCL and assessed the efficacy and tolerability of the main first-line treatments together with the subsequent therapy options and outcomes. We also identified the infections associated with the course of the disease. HCL occurs more frequently in males, and the median age of diagnosis is 52 years [14]. Our patient cohort showed characteristics that were similar to the previously reported literature with a median age of 49 years and a male/female ratio of approximately 7:1.

In our cohort, we identified peripheral and/or visceral lymphadenopathy in 27 patients (38%). In the most recent consensus guidelines for the diagnosis/management of patients with classic HCL, performing imaging studies for the detection of, e.g., lymphadenopathy was optional [15]. It was recommended that these procedures be reserved for patients in a clinical trial or those with associated symptoms referable to these systems [15]. We did not routinely perform imaging studies for all of our patients, but rather only for patients with signs/symptoms for which ultrasonography and/or computerized tomography scanning were indicated. Furthermore, patients with HCL may develop infections (e.g., tuberculosis) or secondary primary tumors [16], where lymphadenopathy could be observed in the course of the disease. Most probably, in some of the patients, these enlarged lymph nodes are reactive or are due to other conditions, including infections.

After the introduction of the purine analogues, the treatment algorithm of HCL evolved greatly [5]. Before that, splenectomy and IFN-α were the mainstays of the treatment [3,4]. The management of HCL in Turkey also changed from the first years of the 2000s onwards with the availability of 2-CdA in the country, and prior to that time, patients with HCL were receiving mainly INF-α and splenectomy upfront. Thereafter, 2-CdA became the standard choice of treatment for most patients with HCL. Earlier data indicate that patients who were treated with first-line splenectomy were usually younger than patients receiving IFN-α. Both splenectomy and IFN-α have been associated with favorable clinical and hematologic responses. However, the median survival with these treatment modalities was approximately 4 years [3,4]. These earlier findings were confirmed by our results indicating high but not durable ORRs with first-line splenectomy and IFN-α. We observed relapse rates as high as 70% and 50% in patients treated with splenectomy and IFN-α, respectively.

With the upfront-usage of 2-CdA more responses have been reported to be higher and durable. In line with the literature, we only noted 16% R/R disease and a median treatment-free period of 23 months in our patients treated upfront with 2-CdA. This was quite similar to what was reported by Saven et al. [17], demonstrating a relapse rate of 26% after a median follow-up of 29 months. However, with longer follow-up, relapse rates tended to rise to 40% among patients who received 2-CdA upfront [18].

Twenty-seven patients of our cohort (41%) had to be given a second-line treatment for R/R disease after a median TTNT of approximately 1 year. This was consistent with the findings of Zinzani et al. [19], who found that nearly 44% of patients relapsed after a median of 2.7 years. In our cohort the median TTNT was shorter than that observed in the cohort of Zinzani et al. [19], and most probably the reason for this was the higher percentage of upfront purine analogue usage (85/121, 70%) among their patients than ours (31/66, 47%). As expected, patients receiving first-line 2-CdA had significantly lower relapse rates than those treated with INF-α and splenectomy. Second-line 2-CdA treatment in our cohort of patients resulted in excellent response rates (ORR=100%). Relapse was observed in one patient only, who also received second-line 2-CdA. Re-treating relapsed patients with an additional course of purine analogues is a reasonable option. Zinzani et al. [19] recommended repeating the same treatment regimen with purine analogues in relapse settings, although changing to a different purine analogue might yield a better result. Unfortunately, 2-CdA is the only approved and available purine analogue in Turkey, so we did not have the opportunity of using other purine analogues like pentostatin in patients who relapsed after 2-CdA.

Although the PFS rate with 2-CdA was significantly higher than those with INF-α and splenectomy, we found similar OS rates with all three upfront treatment modalities. Most probably one of the reasons for this is the relatively short follow-up duration of our study. In addition to that, since most of the patients with HCL may relapse during follow-up, sequentially re-challenging the previous successful treatment(s) as well as the administration of alternative effective agents might have a positive impact on OS. In our cohort, switching to other potent therapies at relapse could be another reasonable explanation for the comparable OS rates between these 3 first-line treatment groups.

A median follow-up duration of 57 months might not be enough to show OS benefit in patients with chronic leukemias such as HCL. After four lines of treatments with a median follow-up of approximately 6 years, 10 patients died and 2 were lost to follow-up, giving an OS rate of 83%, which was consistent with the OS rate of 87% that was reported in the article by Zinzani et al. [19].

One of the most important clinical problems in patients with HCL is the development of severe and sometimes life-threatening infections [20]. Gram-positive and gram-negative organisms, Aspergillus, and other fungi are the most common pathogens [21], but tuberculosis [22] and herpes zoster [23] can be observed, as well. In our patient cohort, by far bacterial and fungal infections were the most common, and 3 patients died due to severe bacterial infection, sepsis, and IPA. We also had 6 patients with 7 episodes of tuberculosis infection. Two patients had tuberculosis and HCL at diagnosis, and 5 episodes occurred during/after anti-HCL treatment. Tuberculosis is an important issue since it can lead to the misdiagnosis of patients for HCL relapse [22]. Thus, when a patient with HCL presents with fever, tuberculosis should always be kept in mind, especially where tuberculosis is endemic. We had 2 patients with herpes labialis and 1 patient with influenza A virus subtype H1N1 infection, which has also been documented in earlier reports on patients with HCL [24].

## CONCLUSION

In conclusion, with the introduction of the purine analogues, the treatment of HCL has been greatly changed. Among our patient cohort, although ORRs were comparable for first-line 2-CdA, INF-α, and splenectomy, patients with frontline 2-CdA had superior ORRs with more durable responses with a higher PFS rate than splenectomy and INF-α cases. The OS rate of the entire cohort was consistent with the current literature. Infections including tuberculosis were a major problem, which caused morbidity and mortality.

Although purine analogues improved the CR rates and PFS, there is still much progress to be made with regard to OS and R/R disease. In that sense, the addition of monoclonal antibodies to purine analogues or incorporation of new target-oriented therapeutic agents such as BRAF, Bruton’s tyrosine kinase, or phosphoinositide 3-kinase inhibitors into treatment regimens might help change the prognosis of the disease further, especially for younger patients and for those who would poorly tolerate the current therapy options.

## Figures and Tables

**Table 1:**
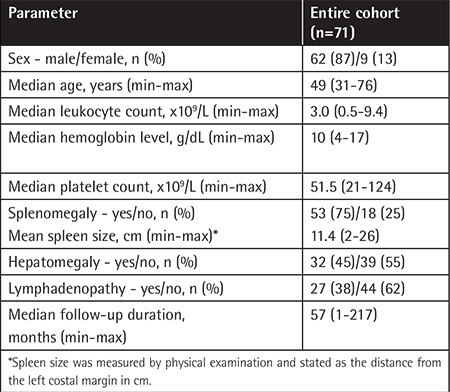
The baseline characteristics of the patients.

**Table 2:**
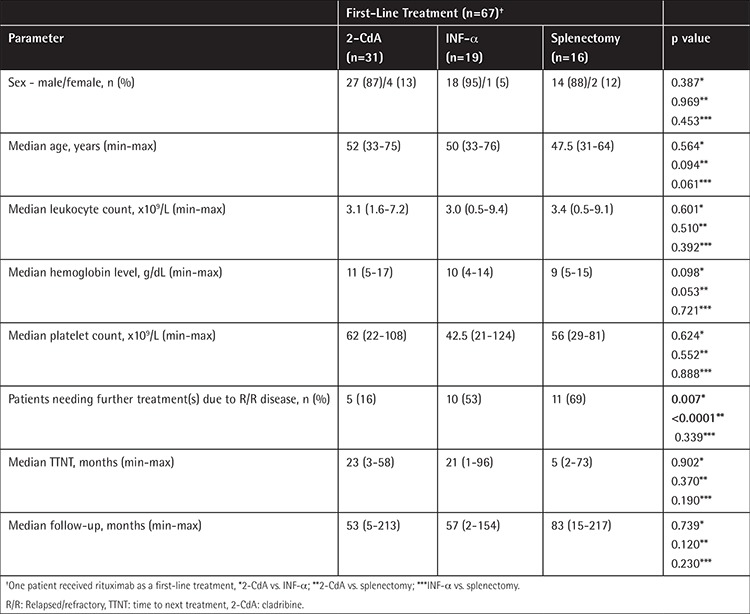
Patient characteristics and treatment outcomes in patients with 3 first-line treatment options.

**Table 3:**
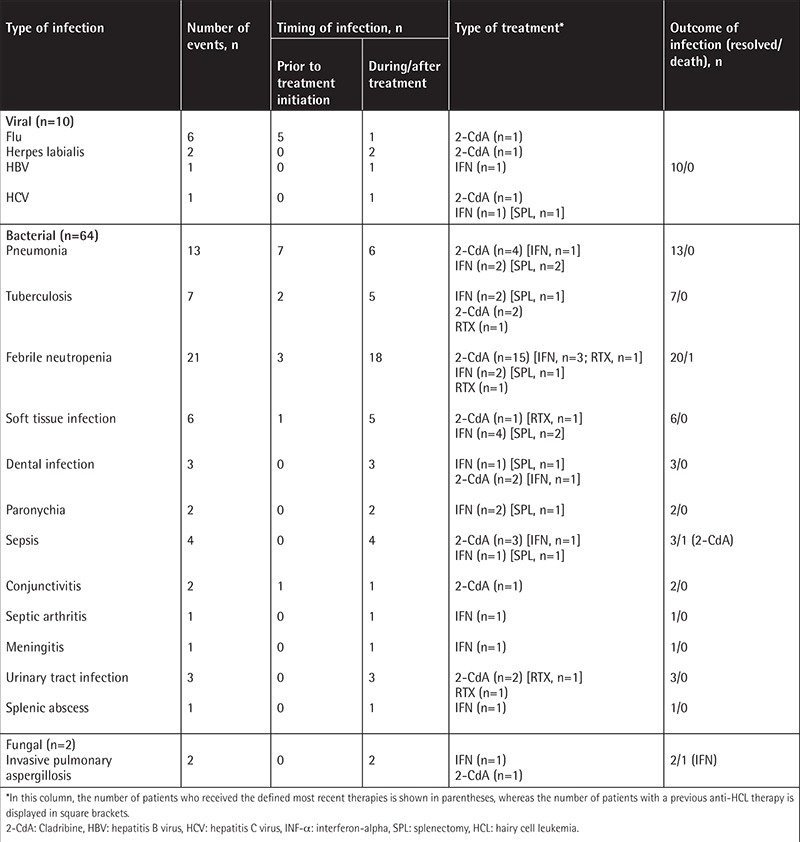
The distribution of infections among the patient cohort.
